# Magnetic Resonance Water Proton Relaxation in Protein Solutions and Tissue: T_1ρ_ Dispersion Characterization

**DOI:** 10.1371/journal.pone.0008565

**Published:** 2010-01-05

**Authors:** Enn-Ling Chen, Raymond J. Kim

**Affiliations:** 1 Duke Cardiovascular Magnetic Resonance Center, Duke University Medical Center, Durham, North Carolina, United States of America; 2 Department of Medicine, Duke University Medical Center, Durham, North Carolina, United States of America; 3 Department of Radiology, Duke University Medical Center, Durham, North Carolina, United States of America; National Institute on Drug Abuse, National Institutes of Health, United States of America

## Abstract

**Background:**

Image contrast in clinical MRI is often determined by differences in tissue water proton relaxation behavior. However, many aspects of water proton relaxation in complex biological media, such as protein solutions and tissue are not well understood, perhaps due to the limited empirical data.

**Principal Findings:**

Water proton T_1_, T_2_, and T**_1ρ_** of protein solutions and tissue were measured systematically under multiple conditions. Crosslinking or aggregation of protein decreased T_2_ and T**_1ρ_**, but did not change high-field T_1_. T**_1ρ_** dispersion profiles were similar for crosslinked protein solutions, myocardial tissue, and cartilage, and exhibited power law behavior with T**_1ρ_**(0) values that closely approximated T_2_. The T**_1ρ_** dispersion of mobile protein solutions was flat above 5 kHz, but showed a steep curve below 5 kHz that was sensitive to changes in pH. The T**_1ρ_** dispersion of crosslinked BSA and cartilage in DMSO solvent closely resembled that of water solvent above 5 kHz but showed decreased dispersion below 5 kHz.

**Conclusions:**

Proton exchange is a minor pathway for tissue T_1_ and T**_1ρ_** relaxation above 5 kHz. Potential models for relaxation are discussed, however the same molecular mechanism appears to be responsible across 5 decades of frequencies from T**_1ρ_** to T_1_.

## Introduction

Image contrast in clinical MRI is often determined by differences in tissue water relaxation behavior. Although the observed properties of proton relaxation in homogeneous liquids such as pure water, ethanol, and glycerol have been successfully explained by the theory of Bloembergen, Purcell, and Pound (BPP) [1], the mechanism of water relaxation in more complex environments such as tissues is still highly speculative. In part to gain insight into tissue relaxation, many studies have evaluated the relaxation characteristics of protein solutions, since for most tissue, relaxation behavior is dominated by the water-macromolecule interaction [2]. However, few studies have attempted to systematically investigate the relationship between the physico-chemical properties of macromolecules and bulk water relaxation, and there are diverse hypotheses concerning the mechanism of water proton relaxation in protein systems—perhaps due to the limited empirical data.

Of particular interest has been the character of the magnetic field dependence (dispersion) of relaxation in these protein systems. Prior investigations have shown that solutions of immobile proteins have spin-lattice relaxation dispersion characteristics similar to that of various soft tissues [3], [4]. Most of these studies measured T_1_ at low field (<20 MHz) or T**_1.ρ_** (spin-lattice relaxation time in the rotating frame, which is measured at B_1_ field strength [5], [6]) since it is known that water proton T_1_ at high field is insensitive to significant protein structural changes such as the addition of crosslinks [7]. Rationale for the improved sensitivity of low-field dispersion to detect protein or tissue structural changes includes arguments concerning the long correlation times of motion (τ_c_) in systems containing large macromolecules [8]. For instance, T**_1ρ_** will presumably be sensitive to motion with τ_c_ on the order of tens of µsecs to msecs, depending on the achievable RF power and proton solvent linewidth, respectively. However, the determination of motional correlation times—whether single, multiple, or even a continuous distribution—requires assumptions about the characteristic shape of the spectral density function. More recently, several investigators have suggested that conventional, BPP-type relaxation theory is inadequate to explain the low field dispersion behavior of solutions of immobilized proteins or tissues, the implications obviously relating to the validity of previous analyses of molecular motion in these systems [6], [9], [10]. For example, Brown and Koenig proposed that the observed low-field dispersion of T_1_ and T**_1ρ_** of tissue water protons is unrelated to a specific correlation time but rather is due to a field dependence of magnetization transfer between water protons and solid-state broadened protein protons [6]. In any case, further data relating specific structural and/or chemical properties of various tissues and protein solutions with properties of water relaxation will be essential to clarify the contributing processes that lead to tissue water relaxation.

In this study, the T_1_, T_2_, and T**_1ρ_** dispersion of solvent protons in solutions of Bovine Serum Albumin (BSA) were evaluated in detail under conditions of varying crosslink density of proteins, pH, solvents, methylation of proteins, and B_0_ field strength. The results were compared with a similar evaluation of myocardial tissue and cartilage. In addition, the T**_1ρ_** dispersion profiles of both BSA solutions and tissue were analyzed for simple power law or BPP model characteristics. There were two aims: first, to provide data relating water relaxation in protein solutions and tissue to variations in macromolecular environment and structure, and second, to evaluate molecular models of tissue water relaxation using T**_1ρ_** dispersion analysis.

## Materials and Methods

### Experimental Preparation: BSA

Relaxation characteristics were studied using fraction V albumin, which is a mixture of different molecular weight BSA (Sigma Chemical), chromatographically purified monomer BSA (98% pure, Sigma), and dimer BSA (95% pure, Sigma).

#### Crosslinking

Variations in BSA crosslink density were produced by reacting 10% (1.5 mM) or 20% (3.0 mM) solutions of BSA with different amounts of glutaraldehyde (from 10 to 200 mM GA). In order to control for changes in small solute (i.e. various forms of unreacted GA), a series of BSA samples were reacted with GA, at concentrations of 10 mM to 60 mM, and then dialyzed (3.5 kDa cutoff) in excess distilled and deionized H_2_O. Grade I GA (50% aqueous solution of pure monomeric GA, stored at −20° C, Sigma) was used for all experiments at 4.7 Tesla and for experiments at 2 Tesla with BSA monomers and dialyzed samples. Grade II GA (25% aqueous solution of monomeric and small quantities of polymeric GA, 25° C, Sigma) was used for fraction V BSA experiments at 2 Tesla. Care was taken to maintain consistent reaction times (≥8 hrs for all experiments) before NMR measurements were obtained.

#### Validation

**Figure 1:**
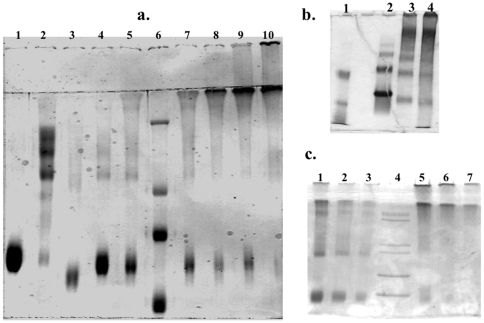
Analysis of bovine serum albumin (BSA) samples by polyacrylamide gel electrophoresis (PAGE). Gels are 7.5% polyacrylamide (PA) with stacking gel of 2% PA and stained with Coomassie Blue. **(a)** SDS-PAGE: Samples were incubated at 95° C in denaturing sample buffer (containing β-mercaptoethanol and SDS), and each lane was loaded with 10 mg of protein and run with SDS in the buffer. Lane 1: BSA monomer; lane 2: BSA dimer; lane 3: methylated BSA (Sigma Chemicals); lanes 4, 5, 7, 8, 9, 10 contain 10% BSA monomers crosslinked with increasing [GA]: 0.1%, 0.2%, 0.3%, 0.4%, 0.5%, 0.6% GA respectively; lane 6: molecular weight marker (229,126,80,48 kDa). **(b)** Non-denaturing PAGE. Lane 1: molecular weight marker (85, 50, 35 kDa); lane 2,3,4 are fraction V BSA with 0.0%, 0.4%, and 0.5% GA respectively. **(c)** Non-denaturing PAGE: Lanes 1–3: methylated BSA (Sigma Chemicals) with decreasing amounts (5, 2.5 and 1.25 ug) of loaded protein; lane 4: molecular weight marker (200, 116, 97, 66, 55 kDa); lanes 5–7: 10% fraction V BSA reacted with 0.4% GA in decreasing amounts (5, 2.5, and 1.25 ug) of protein loaded. See text for details.

Samples of BSA reacted with varying quantities of GA were analyzed with polyacrylamide gel electrophoresis (PAGE) to document progressive increases in BSA molecular weight with increases in [GA]. A standard SDS (Sodium Dodecylsulfate) reducing buffer (Fig. 1a) or a non-denaturing buffer without SDS (Fig. 1b and 1c) was used. Note in lane 1 of figure 1a that purified monomer BSA migrates to a single band near 70 kDa. Lane 2 of figure 1a shows that BSA dimers can exhibit multiple bands. Lanes 4,5, and 7–10 clearly demonstrate that reactions with increasing quantities of GA resulted in the increase not only in the molecular weight of the largest species detected but also in the relative amount of larger to smaller species of BSA. Specifically, as [GA] increases from lane 4 to 10, the monomer band becomes fainter while higher molecular weight species (first BSA in the dimer range, then BSA between 250 and 300 kDa, then BSA polymers that cannot migrate past the 2% stacking gel) become stronger. Figure 1b demonstrates that non-crosslinked, fraction V BSA (lane 2) is composed of a mixture of albumin with different molecular weights unlike purified monomer BSA. The strongest band, however, migrated to a molecular weight of around 70 kDa, similar to monomer purified BSA. In contrast, the strongest bands in the crosslinked, fraction V BSA samples were above 200 kDa. Lanes containing methylated BSA will be described below in Results.

#### pH dependence

Since pH can significantly affect proton chemical exchange rates [11], a subset of BSA relaxation measurements were performed at both pH 5.5 and 7.0. After hydrochloric acid (1 N) was added to the BSA solutions, pH was measured at room temperature using a Mettler pH meter.

#### DMSO solvent

The importance of chemical exchange effects on relaxation may be studied by substituting dimethyl sulfoxide (DMSO) solvent for water since DMSO does not have exchangeable protons. A 10% solution of crosslinked BSA (60 mM GA) was dialyzed (3.5 kDa cutoff) twice against excess DMSO (ACS Reagent, Sigma) at room temperature for 24 hours each. Both the resultant dialysate as well as the original BSA in water solvent were analyzed. The presence of the methyl proton of DMSO and the absence of observable water resonance was confirmed on ^1^H NMR spectra of the dialysate.

#### Methylation

The methylation of BSA in this study refers to the methyl esterification of the carboxyl groups on the BSA molecule (CH_3_-O-BSA). The lyophilized form of methylated BSA (Sigma Chemicals) was dissolved in H_2_O (10% w/v) resulting in a clear solution with neutral pH. Deuterated methyl ester of BSA was synthesized by reacting fraction V BSA with deuterated methanol (Aldrich) (CD_3_OD, 99.8 atom % D) following the protocol of Fraenkel-Conrat [12]. As a control for the synthesis, unlabeled methyl ester of BSA (CH_3_-O-BSA) was synthesized in the same manner except unlabeled methanol (CH_3_OH) was used and then compared to the purchased form of methylated BSA.

### Experimental Preparation: Tissues

#### Myocardium

A 3.5 kg New Zealand White rabbit and a 400 g Sprague-Dawley rat were anesthetized with intravenous sodium pentobarbital (c.a. 50 mg/kg) or diethyl ether respectively. The hearts were rapidly excised and then arrested in cold (4°C) cardioplegic solution containing in mM: NaCl 110, NaHCO_3_ 10, KCl 16, MgCl_2_ 16, and CaCl_2_ 1.2. The posterior papillary muscle of the rabbit left ventricle was then quickly excised keeping the majority of the covering intimal layer intact. It was then dabbed dry and placed in a parafilm-sealed glass tube. After the left ventricular free wall of the rat heart was equilibrated in excess saline (4°C), it was also dabbed dry and placed in a sealed glass tube.

#### Cartilage

Five cubes (4 mm) were cut from a disk of calf patella cartilage which was stored in saline at −20°C. The cubes were thawed, padded dry and equilibrated overnight at room temperature with an excess of one of three different solvents: normal saline (0.9% NaCl in H_2_O), phosphate buffered solutions (100 mM KH_2_PO_4_/K_2_HPO_4_ at pH 9.2, 7.0 and 4.4), and DMSO. After equilibration, the cubes were padded dry and sealed in a glass tube for NMR measurements.

### NMR Measurements

Relaxation measurements at 2 T or 4.7 T were obtained at room temperature with the sample inside a parafilm-sealed 5 mm diameter spherical glass vial using a 4-turn (6 mm diameter) solenoid RF coil. The size and shape of the samples and coils were designed to minimize the spectral linewidth, as well as the RF power required so that the largest range of B_1_ values could be studied. T_1ρ_ was measured with solvent proton (water or DMSO) on resonance and linewidth less than 30 Hz for all protein samples. Solvent proton linewidth was less than 110 Hz for the tissue samples. The T**_1ρ_** pulse sequence consisted of a hard 90° pulse (15 to 50 µs), a 10 µs delay followed by a spinlock pulse on resonance with a 90° phase offset, and then a 100 µs delay and acquisition. The sequence was repeated with step changes in spinlock pulse duration (from 5 msec to approximately 4-fold T_2_). T_1_ was measured using inversion recovery, and T_2_ was measured using single Hahn spin echoes. At least 11 step changes were used for all T_2_ and T_1ρ_ measurements, and at least 21 step changes for all T_1_ measurements. As T_2_ values varied widely, the upper range of TE also varied and was individually adjusted depending on the signal received (TE ranged from 2 msec to approximately 1–2 fold T_2_). For T_1_, T_2_, and T_1ρ_ measurements, the repetition time (TR) was always at least 5×T_1_.

### Data Analysis

T_2_ and T**_1ρ_** relaxation curves were first plotted in semi-log scale to determine the presence of non-single exponential behavior. Relaxation data of all samples except for rabbit papillary muscle appeared single exponential within the time resolution of the NMR experiment. T_1_, T_2_, and T**_1ρ_** relaxation times were then obtained by fitting magnitudes of spectral peaks to two-parameter single exponential functions. All relaxation time values were calculated from a one-time measurement. The T**_1ρ_** dispersion data of both BSA and tissue samples were analyzed for simple power law or BPP model characteristics using equations of the form:
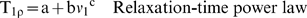
(1)

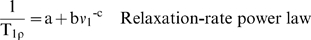
(2)

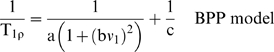
(3)where ν_1_ = γB_1_/2π, and a, b, and c are dispersion parameters whose values are determined by the fitting algorithm. The relaxation times and dispersion parameters were obtained via non-linear least squares fit of the data using the Marquardt-Levenberg algorithm (IDL, Research Systems, Inc.). Convergence occurred when the relative decrease in chi-square between iterations was smaller than 0.01%. Up to 100 iterations were performed before determining a failure to converge.

## Results

Table 1 summarizes the relaxation times and T**_1ρ_** dispersion characteristics for all samples. The standard error of the estimate for the relaxation time curvefits were on average less than 1.0% of the calculated relaxation time values for all protein samples and less than 2.2% for tissue samples. Unless specifically reported, pH was not measured.

**Table 1:** Solvent Relaxation Parameters in Various Protein Solutions and Tissues.

Sample Group	Sample Description	T_1_ (ms)	T_2_ (ms)	T_2_/T_1_	% change in T_1ρ_ (1–30 kHz)		
					1–5 kHz	5–10 kHz	10–30 kHz
**BSA (Fraction V) in H_2_O (2T)**	10% BSA, 0mM GA, pH 5.5	1650	473.2	0.287	83.29	6.72	9.99
	10% BSA, 0mM GA, pH 7.0	1222	338.0	0.277	85.78	6.06	8.16
	10% BSA, 20mM, GA	1535	392.0	0.255	69.70	8.95	21.36
	10% BSA, 40mM GA	1632	347.0	0.213	52.94	10.80	36.25
	10% BSA, 80mM GA	1520	167.6	0.110	17.23	22.91	59.86
	10% BSA, 100mM GA	1557	136.5	0.088	25.49	21.22	53.29
	20% BSA, 200mM GA	766	58.7	0.077	17.96	23.78	58.26
**BSA (Monomers) in H_2_O (2T)**	10% BSA, 0mM GA	1873	256.8	0.137	64.32	13.11	22.57
	10% BSA, 20mM GA	1793	249.0	0.139	81.74	10.10	8.16
	10% BSA, 40mM GA	1746	229.0	0.131	44.65	17.20	38.16
	10% BSA, 60mM GA	1656	206.7	0.125	14.54	18.02	67.44
	10% BSA, 80mM GA	1559	155.4	0.100	13.32	22.53	64.15
	10% BSA, 100mM GA	1617	136.8	0.085	20.07	22.02	57.90
	10% BSA dimer	1674	275.5	0.165	74.05	16.99	8.96
**BSA (Fraction V) in H_2_O (4.7T)**	10% BSA, 0mM GA	1767	233.1	0.132	63.63	21.89	14.48
	10% BSA, 20mM GA	1715	257.0	0.150	56.84	18.78	24.38
	10% BSA, 40mM GA	1695	204.0	0.120	25.93	18.47	55.60
	10% BSA, 60mM GA	1703	168.0	0.099	21.49	18.78	59.73
	10% BSA, 80mM GA	1833	109.3	0.060	21.44	20.89	57.67
	10% BSA, 200mM GA	1852	82.6	0.045	20.55	19.03	60.42
**10% BSA (Fraction V), 60mM GA (2T)**	undialyzed	1453	146.3	0.101	19.95	20.89	59.16
	dialyzed in DMSO	1121	54.0	0.048	12.42	21.43	66.15
	dialyzed in H2O	1542	52.4	0.034	23.43	22.65	53.92
**10% BSA (methylated) (2T)**	methylated BSA (Sigma)	1999	190.3	0.095	42.38	17.59	40.04
	methylated BSA (synthesized)	2080	275.3	0.132	47.13	14.27	38.60
	^2^H-methylated BSA (synthesized)	2313	272.8	0.118	41.84	11.91	46.24
**4.7T**	rabbit myocardium	1396	44.0	0.032	26.80	20.83	52.37
**2T**	rat myocardium	1131	50.5	0.045	19.84	20.37	59.79
**Cartilage (2T)**	in saline	847	64.2	0.076	26.10	24.61	49.29
	in DMSO	351	6.4	0.018	11.19	20.56	68.25
	in phosphate buffer pH 9.2	1047	27.7	0.026	18.48	21.98	59.54
	in phosphate buffer pH 7.0	987	48.1	0.049	26.25	24.40	49.35
	in phosphate buffer pH 4.35	953	40.3	0.042	23.33	26.11	50.56

BSA = Bovine serum albumin, GA = Glutaraldehyde, DMSO = Dimethyl sulfoxide.

### BSA Samples

**Figure 2:**
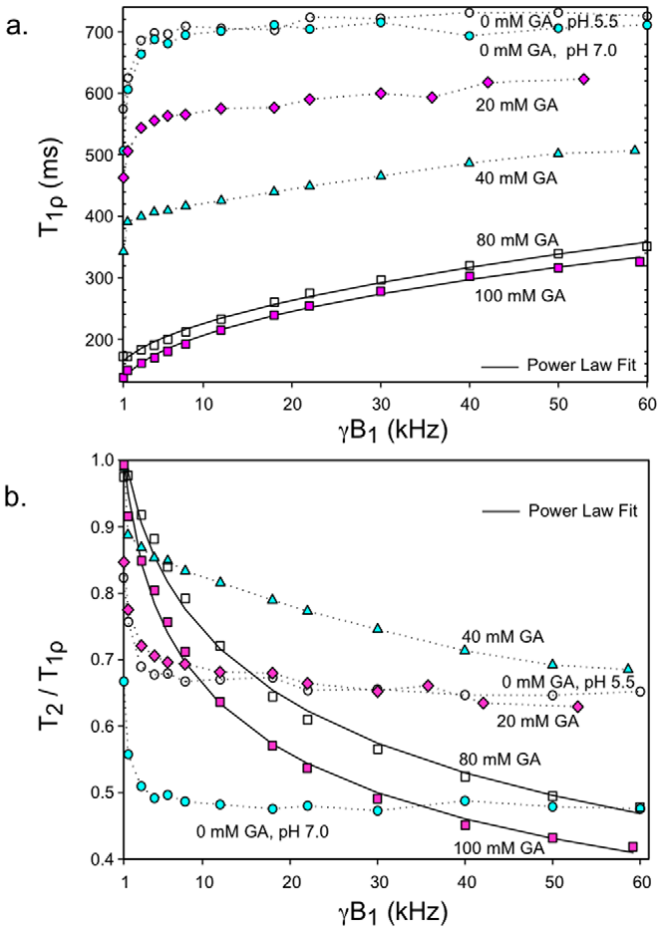
T_1ρ_ dispersion characteristics of various protein solutions. **(a)** T**_1ρ_** of water protons in solutions of 10% BSA (fraction V) versus B_1_ field strength at various glutaraldehyde (GA) concentrations. Samples were evaluated at 2T. Uncrosslinked BSA samples were studied at pH 5.5 and 7.0. The data points for 80 and 100 mM GA were fitted to the relaxation-time power law of Eq. [1]. **(b)** T_2_ measurements were incorporated into the data of panel a to show T_2_/T**_1ρ_** ratios as a function of B_1_ field strength. See text for details.

Figure 2a demonstrates two distinct patterns of T**_1ρ_** dispersion for fraction V BSA samples at 2 Tesla. In the absence of crosslinking (0% GA), T**_1ρ_** sharply increased for γB_1_ from 1 to 5 kHz (21% and 34% increase in T**_1ρ_** values for acidic and neutral samples respectively) and then quickly plateaued beyond 10 kHz (<3% increase up to 60 kHz for both acidic and neutral samples). High concentrations of GA (≥80 mM), in contrast, led to smooth and monotonically increasing T**_1ρ_** values from 1 to 60 kHz. For example, T**_1ρ_** increased 25% from 1 to 5 kHz and 53% from 10 to 60 kHz for the sample reacted with 80 mM GA, and the dispersion curves no longer displayed an acute transition zone near 5 kHz. Since BSA reacted with 80 mM GA was a homogeneous liquid and BSA reacted with 100 mM GA was a gel, data in Fig. 2a also demonstrate that gelation, by itself, has little effect on T_1ρ_ dispersion from 1 to 60 kHz.

Figure 2b shows the T_2_/T**_1ρ_** ratio as a function of γB_1_ for the same samples as in Fig. 2a. Note that this ratio approaches unity at 1 kHz γB_1_ as [GA] increases, indicating that highly crosslinked BSA has minimal dispersion (i.e. T**_1ρ_** changes little with B_1_) below 1 kHz. Uncrosslinked samples, on the other hand, had ratios significantly less than 1, pointing to significant dispersion below 1 kHz. The maximum difference in T**_1ρ_** between the acidic and neutral uncrosslinked samples occurred at zero field (measured as T_2_) where the T_2_ of the acidic sample was 40% above that of the neutral sample.

**Figure 3:**
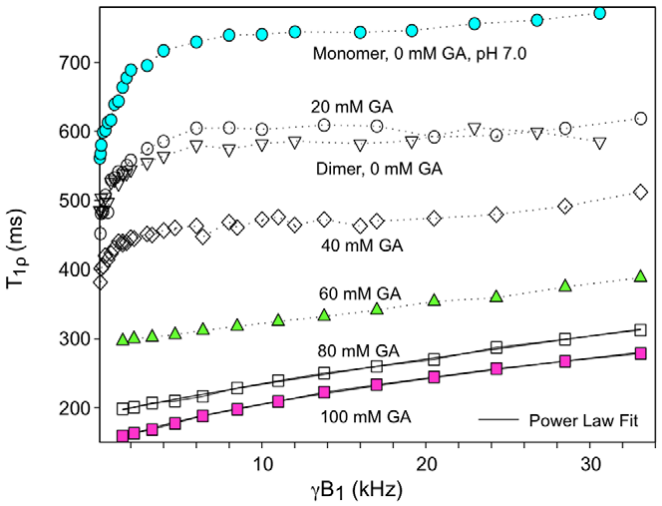
T_1ρ_ dispersion characteristics of BSA solutions derived from purified monomers. T**_1ρ_** of water protons in solutions of 10% BSA (purified monomers) versus B_1_ field strength at various glutaraldehyde (GA) concentrations evaluated at 2T. Data from uncrosslinked purified BSA dimers are also shown. The solid lines represent the fit to the relaxation-time power law of Eq. [1].

Figure 3 shows T**_1ρ_** dispersion plots of purified monomers (or dimer) of BSA rather than fraction V BSA. T**_1ρ_** values for native (0% GA) purified BSA dimers nearly coincided with those of monomers reacted with a low concentration of GA (20 mM) and showed only subtle changes compared to uncrosslinked BSA monomers (see also Table 1). Similar to the dispersion plots of fraction V BSA, purified monomers of BSA treated with increasing [GA] up to 60 mM showed increasing T**_1ρ_** dispersion for γB_1_ beyond 5 kHz. Increasing [GA] above 60 mM did not significantly change the dispersion characteristics of crosslinked monomers of BSA.

**Figure 4:**
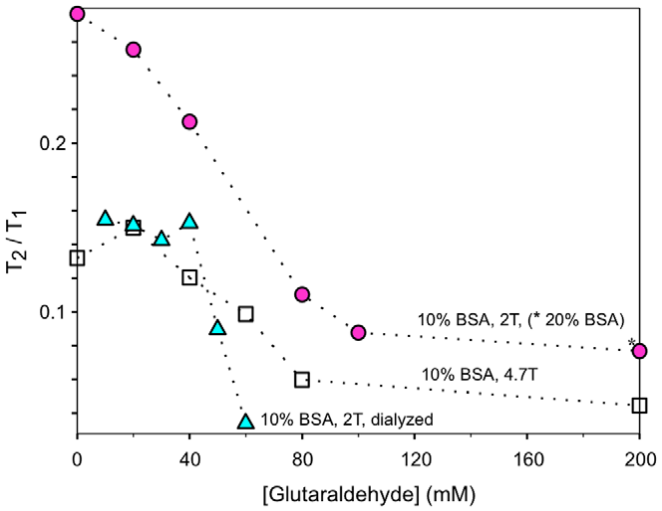
T_2_/T_1_ ratio of BSA solutions reacted with various concentrations of glutaraldehyde. All samples contain 10% BSA (fraction V), except for one sample (*) with 20% BSA. Measurements were performed at both 2 and 4.7 T. See text for details.

Figure 4 plots the T_2_/T_1_ ratio of BSA samples reacted with varying concentration of GA at both 2.0 T and 4.7 T and for dialyzed samples. Although T_2_/T_1_ ratios were found to generally decrease with increasing [GA], there was a transition zone between 40 and 60 mM GA where most of the changes in the T_2_/T_1_ ratios occurred. This range of [GA] was also the transition zone for changes in the shape of T_1ρ_ dispersion seen in figures 2 and 3. T_2_/T_1_ ratios for crosslinked BSA samples dialyzed in excess H_2_O to remove the possible effects of unreacted GA showed a similar dependence on [GA] as undialyzed samples although the transition zone was narrower. T_2_/T_1_ ratios and T**_1ρ_** dispersion of BSA samples obtained at 4.7 T demonstrated a similar dependence on [GA] as those obtained at 2 T (see Table 1).

### Methylation

**Figure 5:**
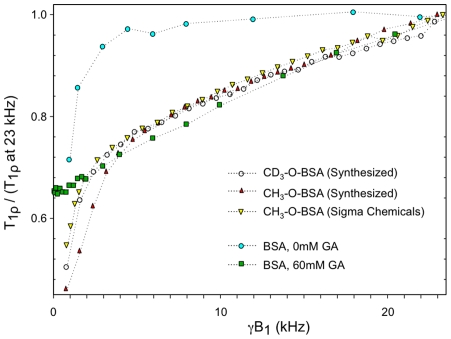
Normalized T_1ρ_ dispersion plots of methylated BSA solutions. Samples were evaluated at 2T. Plots of native and crosslinked BSA (fraction V) are also shown for comparison. Note the similarity of T**_1ρ_** dispersion of methylated BSA with crosslinked BSA above 5 kHz, and the similarity with native BSA below 5 kHz.

Figure 5 compares the T**_1ρ_** dispersion of methylated BSA with crosslinked (60 mM GA) and uncrosslinked BSA. Dispersion curves were normalized to the respective T**_1ρ_** values at the maximum frequency studied (23 kHz) in order to allow direct comparison of dispersion shapes. Figure 5 shows that above 5 kHz γB_1_ methylated BSA had nearly the same T**_1ρ_** dispersion as crosslinked BSA. Below 5 kHz, the dispersion of methylated BSA resembled uncrosslinked BSA. These results were consistent, independent of whether the methyl groups were protonated or 99% deuterated. Deuteron labeling of the methyl groups of methylated BSA led to a minor increase in T_1_ (2313 vs. 2080 ms) and no change in T_2_ values (273 vs. 275 ms) compared with ^1^H-methylated BSA (Table 1). The T**_1ρ_** dispersion of methylated BSA synthesized in the same manner as ^2^H-methylated BSA (see Methods) is shown as a control. The similarity of T**_1ρ_** dispersion of methylated BSA and crosslinked BSA should be interpreted in light of our results in Fig. 1c, which shows that methylated BSA tends to form large aggregates in aqueous solutions (non-denaturing PAGE analysis). Absence of covalent bonding in these aggregates is evidenced by the monomeric appearance of the methylated BSA in denaturing PAGE (Fig. 1a, lane 3).

### Tissues

**Figure 6:**
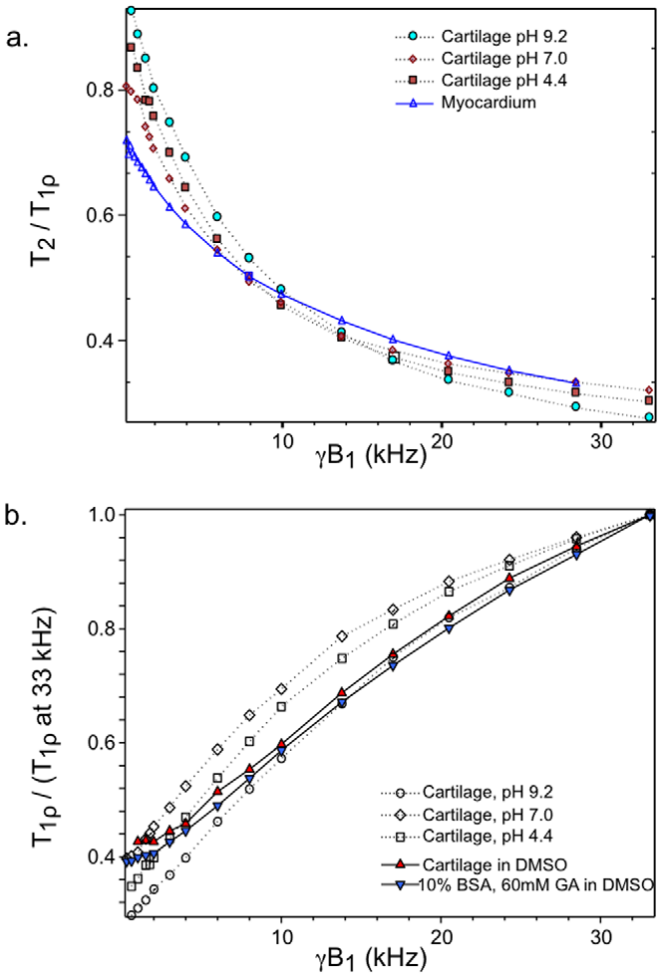
T_2_/T_1ρ_ ratio as a function of B_1_ field strength (a) and normalized T_1ρ_ dispersion plots (b) of various tissue samples. Calf patella cartilage at various pH and rat myocardium samples were evaluated at 2T. Plots of cartilage and crosslinked BSA (fraction V) in DMSO solvent are also shown for comparison in panel b. See text for details.

Figure 6a shows the T_2_/T**_1ρ_** ratio as a function of γB_1_ for cartilage samples as well as for rat myocardial tissue. Note the similarity of these curves with crosslinked BSA in Fig. 2b. Tissue samples uniformly showed smooth monotonically increasing T**_1ρ_** over the entire frequency range studied. For instance, of the total T**_1ρ_** dispersion seen from 1 to 30 kHz for cartilage (pH 7.0, 2 T), 26% occurred between 1 and 5 kHz, 24% between 5 and 10 kHz, and 49% between 10 and 30 kHz. The frequency breakdown in T**_1ρ_** dispersion for crosslinked BSA (10% solution, 100 mM GA) was remarkably similar (25%, 21%, and 53% respectively) as compared to uncrosslinked BSA where generally over 80% of the T**_1ρ_** dispersion occurring from 1 to 30 kHz occurred below 5 kHz (see Table 1). Changes in solvent pH had minor effects on cartilage T**_1ρ_** dispersion, although the T_2_/T**_1ρ_** ratio at 1 kHz γB_1_ for the acidic and basic samples were closer to unity than the neutral sample, indicating less dispersion below 1 kHz for these samples.

To compare the T**_1ρ_** dispersion characteristics of cartilage in water solvent to cartilage in DMSO solvent, normalized dispersion curves are demonstrated in Fig. 6b. For γB_1_ above 5 kHz, the methyl protons of DMSO showed a remarkably similar dispersion curve to that of water protons. Below 5 kHz, cartilage in DMSO displayed minimal T**_1ρ_** dispersion reaching nearly zero slope below 2 kHz. The dispersion curve of crosslinked BSA (10% solution, 60 mM GA) in DMSO nearly coincided with the dispersion curve of cartilage in DMSO from 1 to 33 kHz.

### Dispersion Modeling

**Figure 7:**
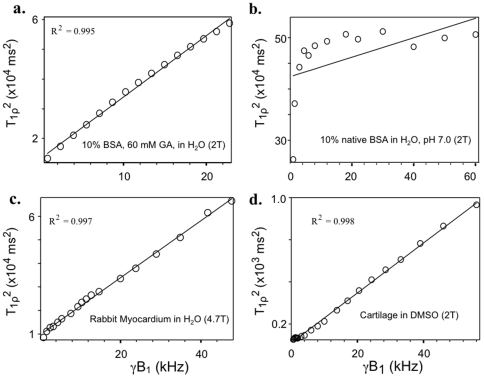
Values of T_1ρ_^2^ are plotted against B_1_ field strength for various samples. **(a)** Crosslinked BSA (fraction V) in H_2_O. **(b)** Native BSA in H_2_O. **(c)** Rabbit myocardial tissue. **(d)** Cartilage in DMSO solvent. See text for details.

As a preliminary test for power law behavior, T**_1ρ_** ^2^ was plotted against γB_1_ for various BSA and tissue samples. Figure 7 demonstrates a near linear relationship between T**_1ρ_** ^2^ and γB_1_ for crosslinked BSA, which did not exist for uncrosslinked BSA. Near linear relationships were also observed for the water protons of rabbit myocardial tissue and the methyl protons of DMSO equilibrated in cartilage. Although some non-linear behavior was present for crosslinked BSA and tissues, especially at lower frequencies, perfect linearity was not expected since it would require the power law exponent to be exactly ½ and the frequency independent component to be negligible.

**Figure 8:**
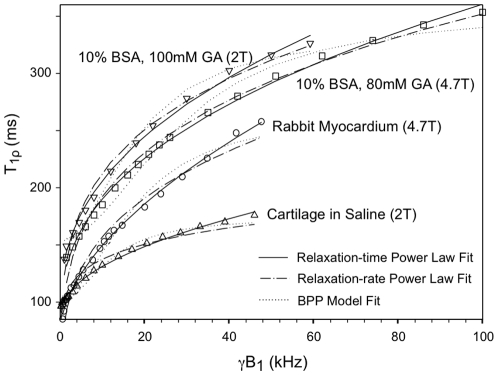
Plots of T_1ρ_ vs. B_1_ field strength are shown for two tissue and two crosslinked BSA samples (fraction V). The lines drawn were fitted using Eqs. [1–3]. Note that relaxation-time power law (Eq. [1]) appears to best fit the data. See text for details.

Figure 8 demonstrates the fits of Eq. [1–3] to representative crosslinked BSA and tissue samples. Table 2 shows that the standard error of the estimate of the fit expressed as a percentage of the T**_1ρ_** value (% SEE) for crosslinked BSA (GA≥60 mM) and tissue samples was 2.2±1.1% for the relaxation-time power law Eq. [1] compared to 10.5±4.8% for the relaxation-rate power law Eq. [2] and 4.0±2.3% for the BPP model Eq. [3]. The improved fit of T**_1ρ_** dispersion using the relaxation-time power law was found to be statistically significant (*P*<0.005 from analysis of variance with Bonferroni correction for both comparisons [13]). Table 2 lists the fitted values of the parameters (a, b, c) for all samples which were successfully fitted to Eq. [1].

**Table 2:** T_1ρ_ Dispersion Analysis of Crosslinked BSA and Tissues.

		γB_1_ Values (kHz)	Model Curve Fit (% Standard Error of the Estimate)	Relaxation-Time Power Law (Eq. [1]) Parameters of Fit		
Sample Groups	Samples	Number of values	Minimum	Maximum	Relaxation-Time Power Law Eq. [1]	Relaxation-Rate Power Law Eq. [2]	BPP Model Eq. [3]	“a” (ms)	“b” (10^−6^×sec^1+C^)	“c” (exponent)
**BSA (Fraction V) in H_2_O (2T)**	10% BSA, 80mM GA	13	0.97	60.0	2.4	6.8	2.9	138	764	0.515
	10% BSA, 100mM GA	13	1.01	59.2	2.1	5.3	5.0	93.8	2247	0.425
	20% BSA, 200mM GA	22	0.14	33.1	2.3	12	3.3	45.7	31.1	0.731
**BSA (Monomers) in H_2_O (2T)**	10% BSA, 60mM GA	13	1.50	33.1	0.4	9.7	0.9	293	3.10	0.993
	10% BSA, 80mM GA	13	1.50	33.1	0.8	17	1.5	187	21.7	0.833
	10% BSA, 100mM GA	13	1.50	33.1	0.9	4.0	1.9	133	298	0.596
**BSA (Fraction V) in H_2_O (4.7T)**	10% BSA, 60mM GA	23	1.05	154	1.2	10	7.3	155	334	0.591
	10% BSA, 80mM GA	19	1.50	100	2.0	4.7	4.5	81.8	2464	0.411
	10% BSA, 200mM GA	23	1.00	154	3.0	9.4	9.5	64.2	785	0.501
**10% BSA (Fraction V), 60mM GA (2T)**	undialyzed	23	0.10	33.1	1.2	8.2	1.9	156	42.7	0.757
	dialyzed in excess DMSO	23	0.10	33.1	2.7	18	1.8	53.2	3.98	0.964
	dialyzed in H_2_O	15	0.79	22.8	1.4	4.8	2.8	91.9	466	0.577
**4.7T**	rabbit myocardial tissue	19	0.59	47.6	2.0	7.2	8.4	74.5	398	0.569
**2T**	rat myocardial tissue	22	0.10	28.5	1.2	12	3.4	68.4	26.9	0.786
**Cartilage (2T)**	in saline	23	0.21	46.0	1.7	6.0	2.9	87.4	509	0.484
	in DMSO	24	0.21	56.0	3.6	17	3.9	9.06	5.87	0.756
	in phosphate buffer pH 9.2	21	0.20	33.1	3.9	17	4.2	26.9	25.8	0.769
	in phosphate buffer pH 7.0	23	0.10	33.1	3.3	13	4.1	51.0	224	0.591
	in phosphate buffer pH 4.35	23	0.10	33.1	4.1	16	4.7	39.6	133	0.635

BSA = Bovine serum albumin, GA = Glutaraldehyde, DMSO = Dimethyl sulfoxide.

## Discussion

### Low Field Dispersion Behavior

In this study we have obtained low field relaxation data of protein solutions and tissue under varying conditions. We show that the T**_1ρ_** dispersion profiles of native BSA solutions are clearly distinct from that of crosslinked BSA. Above 5 kHz γB_1_ the T**_1ρ_** dispersion of 10% native BSA was essentially flat. This result is similar to that of Zhou and Bryant [5] and Koenig and Brown [7] who found minimal T_1_ dispersion of native BSA from 10 to 100 kHz. Below 5 kHz, we found a steep dispersion profile (i.e. T**_1ρ_** changed rapidly with B_1_). Our measured ratio of T_2_ to T_1ρ_ at 1 kHz for native BSA (Fig. 2b) also suggested continued dispersion below 1 kHz. This sharp dispersion from 0 to 5 kHz was sensitive to changes in solution pH and likely is due to chemical exchange of the water protons with the ionizable protons of the protein. As far as we are aware, only Virta et al. [10] and Mäkelä el al. [14] have also measured the T**_1ρ_** of native BSA below 10 kHz. Although Virta et al. observed insignificant T**_1ρ_** dispersion, only two T_1ρ_ data points were obtained below 5 kHz. In contrast, Mäkelä et al. demonstrated similar findings to the current study in that native BSA solutions showed significant T**_1ρ_** dispersion below 5 kHz and were strongly affected by pH.

For our experimental conditions, at least 60 mM GA was required to alter the BSA T**_1ρ_** dispersion to match the smooth monotonically increasing profile that was seen for tissues. Although 40 mM GA was sufficient to form ≈300 kDa BSA oligomers, 60 mM GA formed an additional species of BSA polymers that were unable to migrate through the pores of the 2% stacking gel (Fig. 1a) suggesting at least an order of magnitude increase in molecular weight for this band. The formation of these large BSA polymers was associated with significant T**_1ρ_** dispersion above 5 kHz as well as an abrupt change in the T_2_/T_1_ ratio (fig. 4). This result indicates that a high degree of immobilization is required for protein solutions to accurately model tissue. Not surprisingly, Gore and Brown [15] evaluating proteins with molecular weight range from 1.4 to 483 kDa, and Menon and Allen [16] assessing serum proteins from 69 to 725 kDa found these protein solutions to be poor models for tissue relaxation behavior. Increasing GA above 80 mM, which obviously led to macromolecular structural changes since gelation occurred between 80 and 100 mM GA, did not lead to further changes in dispersion profile (Fig. 2–3) or the T_2_/T_1_ ratio even with an increase in [BSA] to 20%. Apparently a plateau is reached whereby further increases in macromolecular crosslinking does not enhance relaxation. Our finding of a plateau for samples with GA/BSA mole ratios greater than 53 (80 mM GA, 10% BSA) is in contrast to the results of Zhou and Bryant [5]. They showed increases in T_1_ relaxation dispersion with increasing concentrations of GA with no sign of plateau even at a mole ratio of 256 (8.25% BSA). Since BSA polymerization is known to be highly sensitive to concentrations of BSA as well as GA [17], their results may relate to poor production of sufficiently large BSA polymers even at high GA concentrations.

It should be noted that the discussion, so far, assumes only intermolecular crosslinking of BSA is important in the observed changes in water relaxation. It is known that BSA is a rather rigid globular protein in its native state and that the addition of intramolecular crosslinks can warp or stiffen it only slightly [18]. Thus, intramolecular crosslinking of BSA is unlikely to affect protein motion significantly or lead to dipolar interactions that will substantially enhance relaxation.

Surprisingly, methylated BSA showed essentially the same T**_1ρ_** dispersion as that of crosslinked BSA above 5 kHz (Fig. 5). The mechanism of this low field relaxation, however, is clearly independent of dipole-dipole interactions of the methyl protons either directly by spin exchange with tightly bound solvent protons or indirectly by spin diffusion with the protein protons. Significant reduction in both the intra- and intermolecular dipolar interactions of methyl protons by the substitution of deuterons for protons had essentially no effect on the T**_1ρ_** dispersion profile. Rather, the similarity of T**_1ρ_** dispersion of methylated BSA and crosslinked BSA can be explained by Fig. 1c. which shows that methylated BSA tends to form large aggregates in aqueous solutions (non-denaturing PAGE analysis). Presumably, the addition of methyl ester side groups to BSA allows nonspecific intermolecular binding of BSA monomers which, similar to crosslinking BSA, slows macromolecular tumbling below a critical threshold. The absence of covalent bonding in these aggregates is evidenced by the single monomer band of methylated BSA in denaturing PAGE (Fig. 1a). Since the methylated BSA solutions contain mixtures of different sized aggregates (Fig. 1c), the sharp dispersion below 5 kHz can be accounted for by the presence of methylated BSA monomers and small aggregates that behave similar to native BSA, and the continued dispersion above 5 kHz is due to large aggregates which are functionally “immobilized” and behave similarly to crosslinked BSA. Thus, the methylated BSA data provide further evidence that differences in the relaxation properties of native and crosslinked BSA are a consequence of the increase in the polymerization of BSA, rather than other effects of GA, such as its attachment as a chemical side group to BSA. Although motion of proton containing side groups, such as methyl groups have been suggested to provide significant relaxation sinks for large proteins [19], [20], their high mobility [19], [21] implies that no significant enhancement of relaxation can be expected below 100 MHz much less in the kHz regime of T**_1ρ_** [20].

### Modeling

For tissue and solutions of sufficiently crosslinked BSA, significant T**_1ρ_** dispersion was seen for the entire range of γB_1_ studied (up to 150 kHz for some samples, Table 2). Our plots of T**_1ρ_** versus γB_1_ were remarkably similar to the plots of T_1_ versus γB_0_ shown by Bottomley et al. [22] for many tissues. Specifically, both T**_1ρ_** and T_1_ dispersion profiles showed a weak field dependence, which was distinct from the T_1_∝ν^2^ relationship expected for magnetic dipolar interactions in simple homogeneous systems (BPP model, see Eq. [3]). Although, the BPP equation can present a concave-down frequency relationship over a local range (Fig. 8), the same relationship cannot occur over an extended range from kHz to hundreds of MHz—5 decades of frequencies from T**_1ρ_** to T_1_—unless multiple or continuously distributed correlation times are assumed [8], [23], [24].

Our T**_1ρ_** dispersion profiles for crosslinked BSA and tissue from 1–100 kHz displayed continuous, monotonic increases with frequency that did not suggest obvious inflections. Likewise, T_1_ tissue dispersion curves from 1–100 MHz summarized by Bottomley et al. [22] and crosslinked BSA dispersion curves from 10 kHz to ∼100 MHz shown by Koenig and Brown [4] (notwithstanding small, local changes in dispersion due to ^14^N-^1^H quadrupole dips) do not show obvious inflections. Similar to Bottomley et al. [22] who found an excellent fit to T_1_ dispersion using the relationship T_1_ = Aν^B^, the T_1ρ_ dispersion data fit well to the simple relaxation-time power law, T_1ρ_ = a+bν^c^ (Eq. [1]), where parameter “a” was added to account for the zero-field offset, T_1ρ_(0) = T_2_. Not only did this equation present a significantly improved fit to the data compared to the relaxation-rate power law (Eq. [2]) and the BPP model (Eq. [3]), it was able to provide a calculated T**_1ρ_**(0) value that closely approximated T_2_. Specifically, the T**_1ρ_**(0)/T_2_ ratio was near unity (1.10±0.32) for the relaxation-time power law, whereas it was significantly higher (*P*<0.001) for the BPP model (1.40±0.40).

The relaxation-rate power law (Eq. [2]) is similar to the Escanye et al. [23] expression 1/T_1_ = Aν^−1/2^+B. This expression was found to adequately fit T_1_ dispersion of mouse muscle from 7–90 MHz and has the advantage that it can be easily interpreted mechanistically as a fast-exchange two-state model. However, this expression cannot account for properties of relaxation at or near zero field where it predicts T_1_(0) to be zero. The relaxation-rate power law demonstrated a poor fit to our T**_1ρ_** dispersion data.

The exponent “c” in the relaxation-time power law was calculated to be 0.66±0.20, 0.68±0.15, and 0.66±0.12 for crosslinked BSA (GA≥60 mM), myocardial tissue, and cartilage, respectively. Neglecting the effects of the T**_1ρ_**(0) offset, these values are higher than the exponent reported by Bottomley et al. [22] for water proton T_1_ dispersion of skeletal muscle (0.42) and heart muscle (0.36). These values, however, are near the exponent reported by Kimmich et al. [20] for ^1^H T_1_ dispersion of either lyophilized or minimally D_2_O-hydrated (16% by weight) proteins and polypeptides (0.74±0.06). Potential relaxation models that account for simple power law field dependence are discussed below.

### Relaxation Mechanisms

#### Protein-associated water

Nearly all models of water relaxation in macromolecular systems consider one or more new groups of protein-associated water with altered motion that contributes to bulk water relaxation. For example, “hydration layers” at the macromolecular interface have been proposed with increased correlation times in order to explain the dispersion data [23]. In the case of T_1ρ_ relaxation data, invariably an additional correlation time is added to the model to account for the low-field regime [8]. However, observations obtained by high-resolution NMR spectroscopy of proteins [25], relaxation dispersion of water ^17^O [26], and paramagnetic spin labeling [27], strongly suggest that surface hydration water is highly mobile with sub-nanosecond residence times. Thus, it is unlikely that models based on distributions of surface water with restricted motional characteristics or the “exchange diffusion” of water molecules to and from a bound hydration layer can explain the relaxation dispersion of protein solutions or tissue.

Later models have focused on a small number of water molecules buried inside proteins, which are clearly distinguished from surface hydration water by their longer residence times [25]. Denisov and Halle [26] report that the internal water molecules of the globular protein, bovine pancreatic Trypsin inhibitor (BPTI), have residence times (τ_RES_) on the order of 10^−8^ to 10^−6^ seconds, whereas the water molecules on the surface of the protein have an average reorientational correlation time of approximately 20 picoseconds. By studying the relaxation behavior of water ^17^O nuclei, the complicating effects of cross-relaxation and hydrogen exchange were avoided, and they postulate that the origin of the water ^17^O relaxation dispersion of BPTI solutions can be explained by a small number of interior water molecules exchanging with bulk water on the submicrosecond time-scale. Although a consensus view is still lacking, our experimental data will be examined considering this model of protein hydration.

#### Dilute globular protein solutions

Similar to our T_1ρ_ data in native and crosslinked BSA solutions, several investigators have shown that the T_1_ dispersion profiles of dilute globular protein solutions are clearly distinct from those of immobilized protein solutions [5], [7]. At least for mobile protein solutions, the dispersion relation is generally Lorentzian, and the dispersion inflection frequency of water ^1^H, ^2^H, and ^17^O nuclei has been shown to correspond to τ_R_, the rotational correlation time of the protein molecule [26], [28], [29]. Thus, the conventional BPP model along with the condition of motional narrowing (ω_1_τ_c_<<1) is apparently applicable in these protein solutions as the effective correlation time of motion, τ_C_, is easily identified with τ_R_. As suggested by Venu et al [28], interior water molecules with residence times greater than τ_R_ (∼6 ns for BPTI) can sense the Brownian motion of the protein molecule, exchange with bulk water, and thereby contribute to the observed relaxation dispersion. The intrinsic relaxation rate of these buried relaxation sinks was explained quantitatively by intramolecular dipole couplings (∼70%) and many intermolecular dipole couplings with BPTI protons (∼30%). Labile protein protons were also thought to make a significant contribution to the observed water relaxation rate. Contributions from direct nuclear Overhauser effect (NOE) cross-relaxation between protein protons and interior or surface water protons were found to be negligible, which is not surprising given the motional narrowing condition [28].

Irrespective of the actual mechanism, the relation ω_1_τ_c_<<1 for the spinlock experiment predicts an essentially flat ^1^H T**_1ρ_** dispersion below ∼1 MHz. As suggested by Hills [30], proton exchange then becomes the remaining relaxation mechanism that is operative in the low-field regime. Our results (Fig. 2–3) show that this is indeed the case. Native BSA solutions exhibited a sharp dispersion profile below 5 kHz that was sensitive to changes in pH and showed a flat dispersion above 5 kHz. Furthermore, the active dispersion range was consistent with the intrinsic proton exchange rates (700–10,000 s^−1^) measured by Liepinsh and Otting from OH and NH groups of several amino acid side chains under physiologic conditions [31]. Our results, therefore, are consistent with the theoretical T**_1ρ_** dispersion of dilute globular protein solutions proposed by Hills [30].

#### Immobilized proteins and tissues

Rotational immobilization of solute protein can be achieved by a chemical [3] or thermal crosslinking reaction [10] or by non-covalent interactions at high (>50% w/w) protein concentrations [9]. For such solutions and biological tissues, it is assumed that the dispersion inflection frequency no longer reflects protein rotation but instead the residence times of long-lived water molecules that are associated with the protein [32]. However, the dispersion curves are not simply scaled, Lorentzian profiles with shifted inflection frequencies, but are distinctly non-Lorentzian [9]. In addition, immobilized protein solutions also exhibit broader temperature T_1_ minimums which are characterized by lower T_2_/T_1_ ratios than expected by conventional BPP-theory unless a distribution of correlation times is assumed [33]. Although many investigators have incorporated various distributions of correlation times to model the non-Lorentzian T_1_ dispersion data [34], [35], it should be recognized that given enough variables, successful fitting of data can occur and may simply represent a convenient parameterization without physical meaning. Packer [36] noted that the weakest assumption of the approach incorporating distributions of correlation times is that all motional processes modulate the same magnetic dipolar interaction strength. A wider distribution of motional correlation times then will predict higher relaxation rates at high field than those observed [3]. Moreover, our finding that a power law relationship also holds for T_1ρ_ dispersion from 1–100 kHz would imply an even larger distribution of correlation times in this model. The appeal of a BPP-type model is that it corresponds to a well-defined mechanism of relaxation, and thus, physically meaningful parameters such as correlation times of motion can be extracted from the relaxation dispersion data. Nevertheless, for immobilized protein solutions and tissues, calculation of mechanistic parameters using BPP-type models—with their inherent assumptions about the nature of the local interactions causing relaxation and the shape of the spectral density function—is likely erroneous.

Furthermore, unlike for mobile protein solutions, abundant evidence exists for direct NOE cross-relaxation between immobilized protein and solvent protons [37]. Bryant et al. [3] have suggested that the longitudinal relaxation of water protons in solutions of immobilized proteins and tissue is due to magnetic coupling of macromolecular protons with water protons and that the magnetic field dependence of the solid component could be transferred at least partially to the liquid component. The simple power law dispersion profiles found for solid protein protons has been explained by intrinsic motions characteristic of protein backbones by Kimmich and Winter [38]. Independent of the mechanism by which protein protons acquire their relaxation field dependence, Zhou and Bryant [5] have proposed that cross-relaxation could then allow “water spins to report a scaled replica” of the relaxation behavior of the solid system. Efficient coupling is required to allow cross-relaxation, and long-lived water molecules buried inside macromolecules (τ_RES_ up to 200 µs for BPTI [39]) could be an important pathway for the magnetization transfer. Long-lived hydration water in junction zones formed by protein crosslinking have also been postulated [40]. In considering this cross-relaxation model, we note that the similarity of our T**_1ρ_** dispersion profiles to that of published T_1_ profiles for immobilized proteins [20] and tissue [22] indicate a common relaxation mechanism is dominant across 5 decades of modulating field strength. The importance of this model is not settled, however, other relaxation models will need to consider dynamic processes that span this large range of frequencies.

An additional mechanism by which magnetization can be transferred from the solid phase to the solvent phase has been suggested by Hills [30]. A three-site model whereby spin diffusion in the solid phase allows spin exchange between non-exchangeable and exchangeable protein protons followed by proton exchange between water and exchangeable protein protons could provide a quantitative interpretation of the relaxation data without the need to invoke special hydration water. Our T**_1ρ_** dispersion profiles of the methyl protons of DMSO for cartilage and crosslinked BSA, however, show strong field dependence throughout the studied range (Fig. 6b). In fact, normalized dispersion profiles for the DMSO solvent samples were nearly identical to the dispersion profiles of the corresponding samples with water solvent above 5 kHz. Since the methyl protons of DMSO are not exchangeable, proton exchange is obviously not necessary for the strong field dependence of DMSO protons in immobilized protein solutions or tissue. In addition, the minor effect of solvent pH on water T_1ρ_ dispersion of cartilage as compared to native BSA solutions suggests that protein immobilization attenuates the contribution of proton exchange to water relaxation (Fig. 2b & 6a). Thus, proton exchange appears to have a minor role on T_1ρ_ dispersion in immobilized protein solutions and tissues above 5 kHz. Interestingly, Mäkelä el al. [14] using some similar sample preparations, drew nearly the opposite conclusion. Namely, they conclude that there is “…a crucial role of proton exchange on R_1ρ_ and R_1ρ_ dispersion in immoblilzed protein solution mimicking tissue relaxation properties.” However, we note that Mäkelä el al. assessed T_1ρ_ over a much narrower range of γB_1_ (1–11 kHz), did not evaluate DMSO solvent samples, and did not study any tissues. The latter is particularly important, since Mäkelä el al. selectively focus on their results from heat-denatured rather than glutaraldehyde cross-linked BSA, postulating that glutaraldehyde treated BSA is a poor model for tissue, albeit without tissue data to support this supposition.

We also note that our T**_1ρ_** dispersion profiles of BSA and tissue samples showed no significant ω_0_ dependence between 86 and 200 MHz (2 T and 4.7 T). Therefore, exchange models that produce ω_1_ dependence because of a resonance offset, δω, between water and labile protein protons or between long-lived protein associated water and bulk water, cannot account for the T**_1ρ_** dispersions measured in this study.

The similarity of our DMSO and water solvent T**_1ρ_** dispersions implies similar molecular mechanisms for relaxation. Long-lived DMSO molecules, if present, should also have comparable residence times to that of water molecules. It is consistent then that Denisov and Halle [26] indicate that buried water molecules have long residence times due to the free energy cost of local protein unfolding rather than due to a full complement of strong hydrogen bonds. In addition, we note that DMSO solvent in crosslinked BSA and cartilage show nearly identical ^1^H-T**_1ρ_** dispersion (Fig. 6b), despite obvious differences in macromolecular content. This result suggests that buried solvent molecules, which presumably function as relaxation centers, lack sensitivity to details of macromolecular structure.

Thus, in summary, the data of the current study suggest the following relaxation mechanisms. For dilute globular proteins, the conventional BPP model appears to be applicable with the effective correlation time corresponding to protein rotation. Proton exchange is an important contributor to the observed water relaxation rate, whereas cross-relaxation between protein protons and water protons is negligible given the motional narrowing condition. For immobilized proteins and tissue, proton exchange appears to be a minor pathway for T**_1ρ_** and T_1_ relaxation above 5 kHz. The data are consistent with special water protons, perhaps located internally, that have enhanced relaxation. The relaxation of these special water protons is possibly due to cross-relaxation with immobilized protein protons, although intra- or intermolecular dipole interactions of these special water protons may also contribute. The smooth monotonic relaxation dispersion across 5 decades of frequencies (from T**_1ρ_** to T_1_) may or may not reflect the relaxation behavior of the solid system, but nonetheless, implies a failure of the simple BPP model.
